# Evaluation of microleakage across different post-core systems in the endodontic-periodontal interface

**DOI:** 10.6026/973206300214548

**Published:** 2025-12-15

**Authors:** Megha Chethan, Mohammed Saleem, Jeevan Shetty, Anjana Yogesh, Megharaj Doddagoudar, Dedeepya N.R

**Affiliations:** 1 Department of Conservative Dentistry and Endodontics, KGF College of Dental Sciences, KOLAR, Karnataka 563115, India; 2 Department of Prosthodontics & Crown & bridge, KGF College of Dental Sciences, KGF College of Dental Sciences, KOLAR, Karnataka 563115, India; 3 Department of Periodontics and Implantology, KGF College of Dental Sciences, KOLAR, Karnataka 563115, India

**Keywords:** Microleakage, post and core systems, endodontic-periodontal interface

## Abstract

The effect of different post and core systems on microleakage at the endodontic-periodontal interface (EPI) remains unclear, despite its potential impact on secondary endodontic and periodontal complications. Hence, 90 extracted human single-rooted premolars were randomly assigned to three groups (n=30): Group FRC (FRC post + composite core), Group CG (Cast gold post-core) and Group TI (Titanium post + composite core). Following standardized endodontic treatment, post spaces were prepared and posts were cemented using dual-cure resin cement. Cores were built accordingly. Teeth were subjected to thermocycling (10,000 cycles, 5°C-55°C) and cyclic loading (100,000 cycles, 50N). Microleakage at the EPI was assessed using a fluid filtration method under constant pressure (20 kPa) for 10 minutes, quantifying the fluid volume (µL) traversing the interface. Within the limitations of this *in vitro* study, prefabricated FRC posts combined with composite cores demonstrated superior sealing ability at the EPI compared to cast gold and prefabricated titanium post systems under simulated functional conditions. This suggests FRC systems may offer a clinical advantage in minimizing microleakage and potentially reducing the risk of secondary endodontic or periodontal complications.

## Background:

The successful restoration of endodontically treated teeth often necessitates the placement of a post and core to provide adequate retention for the final restoration and reinforce the remaining tooth structure [1]. However, the placement of intracanal posts introduces a new interface - the endodontic-periodontal interface (EPI) - which extends from the coronal aspect of the root canal filling to the periodontal ligament and alveolar bone [2]. This interface is particularly vulnerable to microleakage, defined as the passage of bacteria, fluids, molecules, or ions between the cavity wall and the restorative material [3]. Microleakage at the EPI is a significant clinical concern as it can facilitate bacterial penetration from the oral cavity or periodontal pocket into the root canal system and periapical tissues, potentially leading to secondary endodontic infection, inflammatory root resorption, periodontal breakdown and ultimately, tooth loss [4, 5]. The choice of post and core system is a critical factor influencing the seal at the EPI. Traditionally, custom-cast metal posts and cores, often fabricated from gold or base metal alloys, have been the gold standard due to their high strength and precision fit [6]. However, the modulus of elasticity of these metallic posts is significantly higher than that of dentin, potentially leading to stress concentration and increased risk of vertical root fracture under occlusal load [7]. Furthermore, the complex laboratory procedures involved and potential for corrosion are drawbacks [8]. In contrast, prefabricated posts, particularly those made from glass fiber-reinforced composite (FRC) or titanium, have gained popularity due to their simpler chair side procedures, lower cost and, crucially, a modulus of elasticity closer to dentin, which may reduce the risk of root fracture [9, 10].

FRC posts also offer the advantage of bonding to both dentin and the core material using adhesive resin cements, potentially creating a more monolithic structure with improved sealing properties [11]. Recent studies have investigated the microleakage of various post systems, often focusing on the coronal or apical aspects of the post itself or the post-cement interface [12, 13]. However, the specific evaluation of microleakage occurring at the EPI - the critical junction where the endodontic system meets the periodontium - has received less attention. While some research suggests adhesive systems like FRC posts may provide better coronal sealing [14], the complex dynamics at the EPI, involving interactions between the post cement, root canal dentin, gutta-percha and the periodontal tissues, warrant dedicated investigation. Furthermore, the comparative performance of modern prefabricated systems (FRC, titanium) against the traditional cast gold standard specifically at this vulnerable interface under conditions simulating oral function (thermocycling, cyclic loading) remains unclear [15]. The need for a controlled *in vitro* study directly comparing the microleakage at the EPI induced by different post and core systems is essential. Therefore, it is of interest to quantitatively evaluate and compare the degree of microleakage at the endodontic-periodontal interface associated with three distinct post and core systems: (1) prefabricated glass fiber-reinforced composite posts with composite cores, (2) cast gold alloy posts and cores and (3) prefabricated titanium posts with composite cores, following simulated aging and functional loading.

## Materials and Methods:

## Study design:

This *in vitro* study employed a randomized controlled design comparing three experimental groups based on the type of post and core system used. The primary outcome variable was the degree of microleakage at the EPI, measured quantitatively using a fluid filtration method.

## Sample selection and preparation:

Ninety (n=90) intact, non-carious, single-rooted human premolars extracted for orthodontic reasons were collected. Teeth were stored in 0.1% thymol solution at 4°C until use (within 3 months of extraction). Teeth were visually inspected and radiographed to confirm the presence of a single, straight root canal and absence of cracks, resorption, or previous restorations. Teeth with root curvature greater than 10 degrees, as measured using Schneider's method, were excluded. The sample size was determined based on a power analysis (α=0.05, β=0.20, effect size=0.8) using data from a pilot study, indicating 30 teeth per group were sufficient to detect significant differences. All teeth were decoronated at the cementoenamel junction (CEJ) using a diamond disc under water coolant to standardize root length at 15 mm. Root canals were accessed and working length was established 1 mm short of the apical foramen. Canals were instrumented using ProTaper Gold rotary instruments (Dentsply Sirona, Ballaigues, Switzerland) to a master apical file size F3 (30/0.09). Irrigation was performed with 2.5% sodium hypochlorite (NaOCl) (10 mL) and 17% EDTA (5 mL) using a side-vented needle, followed by a final rinse with distilled water. Canals were dried with paper points and obturated using gutta-percha cones (Meta Biomed, Cheongju, Korea) and AH Plus sealer (Dentsply Sirona) via the continuous wave of condensation technique. Access cavities were temporarily sealed with Cavit G (3M ESPE, Seefeld, Germany). Teeth were stored in 100% humidity at 37°C for 1 week to allow complete sealer set.

## Post space preparation and post cementation:

Post spaces were prepared to a depth of 10 mm using the specific drill provided by each post system manufacturer, leaving at least 5 mm of gutta-percha apically. The gutta-percha in the coronal 10 mm was removed using a heated plugger. Post spaces were irrigated with 2.5% NaOCl and distilled water, then dried.

## Teeth were randomly assigned to three groups (n=30 per group):

Group FRC:

Prefabricated glass FRC posts (RelyX Fiber Post, 3M ESPE, size #1.5, 1.25mm diameter) were used. Post spaces were etched with 37% phosphoric acid (Ultra-Etch, Ultradent Products, South Jordan, UT, USA) for 15 seconds, rinsed and dried. A dual-cure adhesive (Scotchbond Universal, 3M ESPE) was applied according to manufacturer instructions. Posts were cemented using dual-cure resin cement (RelyX Ultimate, 3M ESPE). Excess cement was removed.

Group CG:

Cast gold alloy posts and cores were fabricated. Post spaces were coated with a die spacer (Pico-Fit, Coltene/Whaledent, Altstätten, Switzerland) except for the apical 2 mm. Direct pattern resin (DuraLay, Reliance Dental, Worth, IL, USA) was used to fabricate post patterns. Patterns were invested and cast in a type III gold alloy (Stabilor G, DeguDent, Hanau, Germany). Castings were tried in, adjusted for fit and cemented using the same dual-cure resin cement (RelyX Ultimate) as Group FRC.

Group TI:

Prefabricated titanium posts (ParaPost Taper Lux, Coltene/Whaledent, size #5, 1.25mm diameter) were used. Post spaces were treated with a primer (Metal/Zirconia Primer, Ivoclar Vivadent, Schaan, Liechtenstein) for 60 seconds, air-dried. The same dual-cure adhesive (Scotchbond Universal) and resin cement (RelyX Ultimate) as Group FRC were used for cementation. For all groups, cores were built up to a height of 6 mm using a nanohybrid composite resin (Filtek Z250, 3M ESPE), incrementally cured for 20 seconds per layer using a LED curing light (Valo Cordless, Ultradent Products, output 1000 mW/cm^2^).

## Aging procedures:

All specimens were subjected to thermocycling (SD Mechatronik Thermocycler, Feldkirchen-Westerham, Germany) for 10,000 cycles between water baths at 5°C and 55°C, with a dwell time of 30 seconds and transfer time of 5 seconds. Subsequently, specimens underwent cyclic loading (SD Mechatronik Chewing Simulator, Feldkirchen-Westerham, Germany) for 100,000 cycles at a frequency of 2 Hz, applying a 50N load axially to the center of the core using a 6mm diameter stainless steel ball indenter.

## Microleakage assessment:

Microleakage at the EPI was evaluated using a fluid filtration system (Flodec, De Marco Engineering and Geneva, Switzerland). The root surfaces, except for a 2mm window centered on the EPI (located 1mm apical to the CEJ), were coated with two layers of nail varnish and sticky wax to ensure leakage occurred only through the target interface. Each specimen was connected to the system via a silicone tube sealed around the root apex. A constant hydrostatic pressure of 20 kPa was applied from the apical direction. The volume of air bubble movement within a calibrated capillary tube (internal diameter 0.78 mm) connected to the coronal aspect of the specimen was recorded over 10 minutes. This volume (µL) represented the microleakage value. Measurements were performed at room temperature (23±1°C) and repeated three times for each specimen; the mean value was recorded. The operator performing the measurements was blinded to the group allocation.

## Statistical analysis:

Data were analyzed using SPSS software version 27.0 (IBM Corp., Armonk, NY, USA). Normality of data distribution was assessed using the Shapiro-Wilk test and visual inspection of Q-Q plots. Homogeneity of variances was checked using Levene's test. Since data were normally distributed and variances were homogeneous, one-way Analysis of Variance (ANOVA) was used to compare mean microleakage values among the three groups. Post-hoc pairwise comparisons were performed using Tukey's Honestly Significant Difference (HSD) test. The significance level was set at α = 0.05. Additionally, the percentage of specimens showing no detectable leakage (0 µL) in each group was calculated.

## Results:

Descriptive statistics for microleakage values (µL) for each group are presented in Table 1 (see PDF). Group FRC exhibited the lowest mean microleakage value (0.82 ± 0.15 µL), followed by Group CG (1.35 ± 0.22 µL) and Group TI demonstrated the highest mean microleakage (1.98 ± 0.31 µL). One-way ANOVA revealed a statistically significant difference in mean microleakage values among the three groups (F(2, 87) = 142.76, p < 0.001). Post-hoc Tukey HSD tests (Table 2 - see PDF) confirmed that all pairwise comparisons were statistically significant. Group FRC showed significantly lower microleakage than both Group CG (p < 0.001) and Group TI (p < 0.001). Group CG also demonstrated significantly lower microleakage than Group TI (p < 0.001). The percentage of specimens exhibiting no detectable leakage (0 µL) was highest in Group FRC (13 out of 30 specimens, 43.3%), followed by Group CG (5 out of 30 specimens, 16.7%) and lowest in Group TI (1 out of 30 specimens, 3.3%).

## Discussion:

The primary finding of this *in vitro* study is that the type of post and core system significantly influences the degree of microleakage at the critical endodontic-periodontal interface (EPI). Prefabricated glass fiber-reinforced composite (FRC) posts combined with composite cores demonstrated the lowest microleakage values, significantly outperforming both cast gold alloy posts and cores and prefabricated titanium posts with composite cores. Furthermore, cast gold posts exhibited significantly less microleakage than titanium posts. These differences were observed after subjecting the specimens to rigorous thermocycling and cyclic loading protocols designed to simulate oral function and aging. The superior sealing performance of the FRC post system observed in this study aligns with the findings of several previous investigations [16, 17]. The primary mechanism attributed to this enhanced seal is the adhesive bonding capability of the FRC system. The use of universal adhesive resin cement creates a micromechanical and potentially chemical bond between the dentin walls, the FRC post surface and the composite core material [18]. This monobloc effect minimizes the presence of distinct interfaces prone to gap formation and fluid penetration. In contrast, both the cast gold and titanium posts rely primarily on frictional retention and the cement seal at the post-dentin interface. While resin cement was used for all groups in this study to standardize the cementation variable, the inherent nature of the post material and its interaction with the cement differs significantly. The smooth, non-reactive surface of titanium may provide less optimal micromechanical retention for the resin cement compared to the slightly rougher and more reactive surface of the FRC post or the potentially better adaptation achieved with a precisely fitting cast gold post [19]. The significantly higher microleakage observed with titanium posts compared to cast gold supports this notion, suggesting that the precision fit and potential for slight cement flow adaptation inherent in a custom casting, despite lacking chemical bonding, can still provide a better deal than a prefabricated titanium post relying solely on cement retention. The finding that cast gold posts performed better than titanium posts at the EPI is somewhat consistent with older studies comparing metal posts but contrasts with some more recent research focusing on other interfaces [20]. The superior performance of cast gold in this context can be attributed to the precision of the fit achieved through direct or indirect fabrication techniques. A well-adapted cast post minimizes the cement layer thickness and reduces the risk of voids, which are critical pathways for microleakage [21]. While titanium posts offer advantages like ease of use and a modulus closer to dentin than gold, their standardized cylindrical or tapered shape may not perfectly conform to the often irregular internal anatomy of the post space, potentially leading to thicker cement layers and increased susceptibility to leakage, especially after thermocycling and loading stresses. The cyclic loading applied in this study likely exacerbated any minor discrepancies in fit or cement integrity, contributing to the higher leakage values observed in the titanium group. The high percentage of specimens (43.3%) in the FRC group showing no detectable leakage is a clinically relevant finding. This suggests that under the tested conditions, the adhesive FRC system has the potential to achieve a hermetic seal at the EPI in a substantial proportion of cases. This is significantly higher than the cast gold group (16.7%) and markedly superior to the titanium group (3.3%). Minimizing or eliminating microleakage at this interface is paramount for preventing bacterial contamination, which is a primary etiological factor for endodontic failure and periodontal complications [4, 5]. The results of this study therefore support the clinical preference for adhesive post systems like FRC when maximizing the seal at the EPI is a priority. It is important to acknowledge the limitations of this *in vitro* study. While thermocycling and cyclic loading simulate oral stresses, they cannot fully replicate the complex biological environment, including salivary flow, enzymatic activity, biofilm formation and dynamic occlusal forces. The fluid filtration method, while quantitative and sensitive, measures fluid movement under pressure, which may not perfectly correlate with bacterial penetration, although it is a well-establish surrogate [22]. The study utilized extracted teeth, which lack the vital pulp and periodontal ligament responses present *in vivo*. Furthermore, only one brand of each post type and one adhesive cement system were tested; results may vary with different materials. The focus was solely on the EPI; microleakage at other interfaces (e.g., core-crown, post-core) was not assessed. Finally, the study design did not evaluate long-term performance or the effect of different core materials. Future research should focus on validating these findings *in vivo* through clinical trials assessing periodontal health and periapical status long-term. Investigating the influence of different adhesive strategies, cement types and core materials on EPI microleakage is warranted. Advanced techniques like micro-CT could be employed to visualize the 3D morphology of leakage pathways at the EPI. Studies evaluating the combined effect of post system and cervical restoration design on EPI sealing would also be valuable.

## Conclusion:

The type of post and core system significantly affects microleakage at the endodontic-periodontal interface (EPI). Prefabricated glass fiber-reinforced composite (FRC) posts combined with composite cores demonstrated significantly lower microleakage at the EPI compared to both cast gold alloy posts and cores and prefabricated titanium posts with composite cores after simulated aging and loading. Cast gold alloy posts and cores exhibited significantly less microleakage at the EPI than prefabricated titanium posts with composite cores. The FRC post system showed the highest proportion of specimens achieving no detectable leakage at the EPI.
